# Mg segregation at inclined facets of pyramidal inversion domains in GaN:Mg

**DOI:** 10.1038/s41598-022-22622-1

**Published:** 2022-10-26

**Authors:** Axel R. Persson, Alexis Papamichail, Vanya Darakchieva, Per O. Å. Persson

**Affiliations:** 1 Thin Film Physics Division, Department of Physics, Chemistry and Biology (IFM), Linköping University, SE-581 83 Linköping, Sweden; 2 Competence Center for III-Nitride Technology C3NiT–Janzén and Terahertz Materials Analysis Center THeMAC, Department of Physics, Chemistry and Biology (IFM), Linköping University, SE-581 83 Linköping, Sweden; 3 Solid State Physics and NanoLund, Lund University, P. O. Box 118, 221 00 Lund, Sweden

**Keywords:** Transmission electron microscopy, Materials science, Structural properties

## Abstract

Structural defects in Mg-doped GaN were analyzed using high-resolution scanning transmission electron microscopy combined with electron energy loss spectroscopy. The defects, in the shape of inverted pyramids, appear at high concentrations of incorporated Mg, which also lead to a reduction in free-hole concentration in Mg doped GaN. Detailed analysis pinpoints the arrangement of atoms in and around the defects and verify the presence of a well-defined layer of Mg at all facets, including the inclined facets. Our observations have resulted in a model of the pyramid-shaped defect, including structural displacements and compositional replacements, which is verified by image simulations. Finally, the total concentration of Mg atoms bound to these defects were evaluated, enabling a correlation between inactive and defect-bound dopants.

## Introduction

With a 3.4 eV wide direct band gap, Wurtzite (WZ) gallium nitride (GaN) constitutes an important semiconductor for a wide variety of applications including optoelectronics, as well as high-frequency and high-power electronics^1–4^. High conductivity and low resistivity Ohmic contacts as well as high power devices based on GaN, require *p*-type doping^5,6^, however, available doping agents are limited to Mg^5^.

Mg is the only effective *p*-dopant in GaN, but due to its high activation energy^7^ it has limited applicability in devices that require high hole-concentrations. A high activation energy further dictates that higher Mg concentration is required to reach the desired doping level, which in turn is known to result in structural defects^8^. These defects typically appear at Mg concentrations around $10^{19}$ ${\mathrm{cm}}^{-3}$, with more defects appearing at higher Mg concentrations^9,10^. Moreover, the substitutional incorporation of Mg onto the Ga site is associated with the formation of Mg-H complexes in metalorganic chemical vapor deposition (MOCVD) grown materials, where H originates from cracking of the H$_\mathrm {2}$ carrier gas^11^. Post-growth annealing is therefore required for the H dissociation and out-diffusion to achieve *p*-type conductivity in GaN^12^. However, at high Mg concentrations the total Mg concentration is higher than the ionized acceptor or free hole concentrations, meaning Mg is only partially incorporating into electrically active sites or the Mg acceptors are partially compensated^13,14^. The whereabouts of the inactive dopants have been discussed and include the formed structural defects.

The most commonly observed structural defect resulting from excessive Mg doping appears as a hexagonal pyramid, where the apex of the pyramid points in the N-polar direction of the GaN matrix^7,8,15–18^. This defect has been studied by transmission electron microscopy (TEM), spectroscopic methods (energy dispersive spectroscopy, EDS and electron energy-loss spectroscopy, EELS) as well as by atom probe tomography (APT)^7,16,18^. Through dark-field imaging and convergent beam electron diffraction (CBED) analysis, the structure of the defect is identified as a WZ pyramidal inversion domain (PID), with polarity inversion in respect to the ambient matrix^18–20^. In Ga-polar GaN, for example, the hexagonal PIDs form with a flat $\{0001\}$ facet up and exhibit a N-polar structure within. The inclined facets can vary in angle and are identified as $\{11\bar{2}n\}$, where commonly *n* = 3^8,13,17,20–22^.

Atomic resolution high-angle annular dark-field scanning TEM (HAADF-STEM) has resulted in an improved understanding of the structure. For example, Iwata et al.^13^ recently showed, by HAADF-STEM imaging, how electrically inactive Mg atoms are trapped at the top $\{0001\}$ facet of the hexagonal PID and its adjacent lattice planes, where they thereby limit the possible acceptor concentration. By comparing atomic column intensities to simulated images obtained through varying the amount of substitutional Mg on the Ga site, a pure Mg layer was confirmed at the $\{0001\}$ facet, while for adjacent atomic planes, Mg substituted for Ga on $\tfrac{\mathrm {1}}{\mathrm {4}}$ of the sites. The simulated structure was modified from a model that was obtained through first principles density functional calculations based on pseudopotentials, performed by Northrup^23^. Atomic layer ordering through the hexagonal pyramid’s $\{0001\}$ facet was identified as Ga–N–Mg–N–Ga, inverting the structure at the $\{0001\}$ facet of the PID, to match the ambient lattice. However, the presence of Mg at that facet only accounts for a portion of the inactive dopants.

In the present investigation we turn the attention to the structure and composition of the hexagonal PIDs’ inclined facets. These have been less studied, however, they constitute a significant portion of the pyramids’ interface to the ambient matrix and their extension leads to the apex of the PID, which is the presumed nucleation point for the defect. Also, a recent publication confirmed through ab initio calculations that Mg atoms in neither facet act as acceptors^24^.

For the investigation, we employ aberration corrected STEM, both in HAADF and annular bright-field (ABF) modes, together with electron energy-loss spectroscopy spectrum imaging (EELS-SI) to better understand the spatial distribution of Mg and the structure of the facets at the atomic level. Also, high-resolution STEM images in combination with image simulations were used to investigate the polarity and positions of the atoms associated with the PIDs.

Finally, we calculate the amount of Mg trapped at the hexagonal PIDs and compare with secondary ion mass spectrometry (SIMS) measurements of the total Mg concentration as well as the amount of active dopants, obtained from measured by capacitance–voltage (C–V) measurements.

## Results

**Figure 1:**
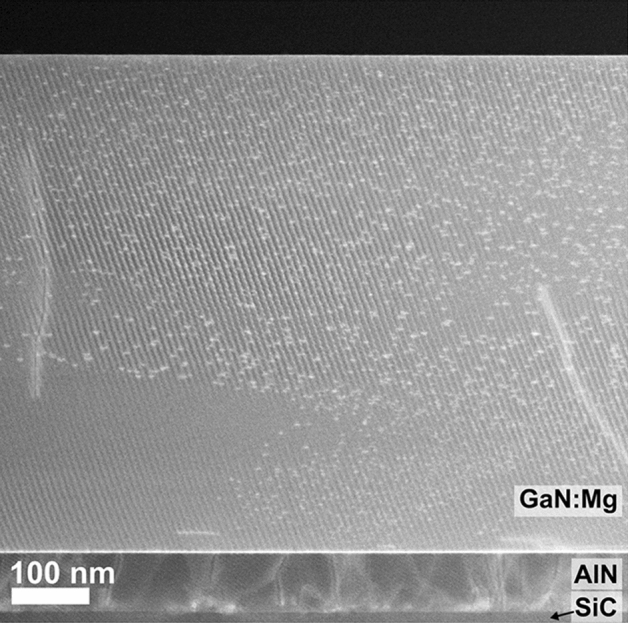
Cross-sectional ADF-STEM overview image of the sample structure: SiC-AlN-GaN:Mg with the substrate at the bottom. The apparent stripes originate from interference between the image pixels and the crystal lattice.

The sample overview (Fig. 1) shows the darker AlN nucleation layer and SiC substrate at the bottom and the GaN:Mg above. Layer thicknesses are 72 ± 3 nm and 625 ± 3 nm respectively. The contrast within the GaN:Mg layer showcases both occasional threading dislocations and hexagonal PIDs (seen as intense spots). The PIDs are randomly distributed and do not correlate with the dislocations. Additionally, the PID size varies, and some regions appear to be free from defects (e.g. bottom left in Fig. 1). These differences are suggested to originate from variations in supply and incorporation of Mg adatoms on the sample surface during the initial stages of growth^7,25^. With increasing thickness, the distribution of defects appears more even, suggesting a steady-state incorporation.

**Figure 2:**
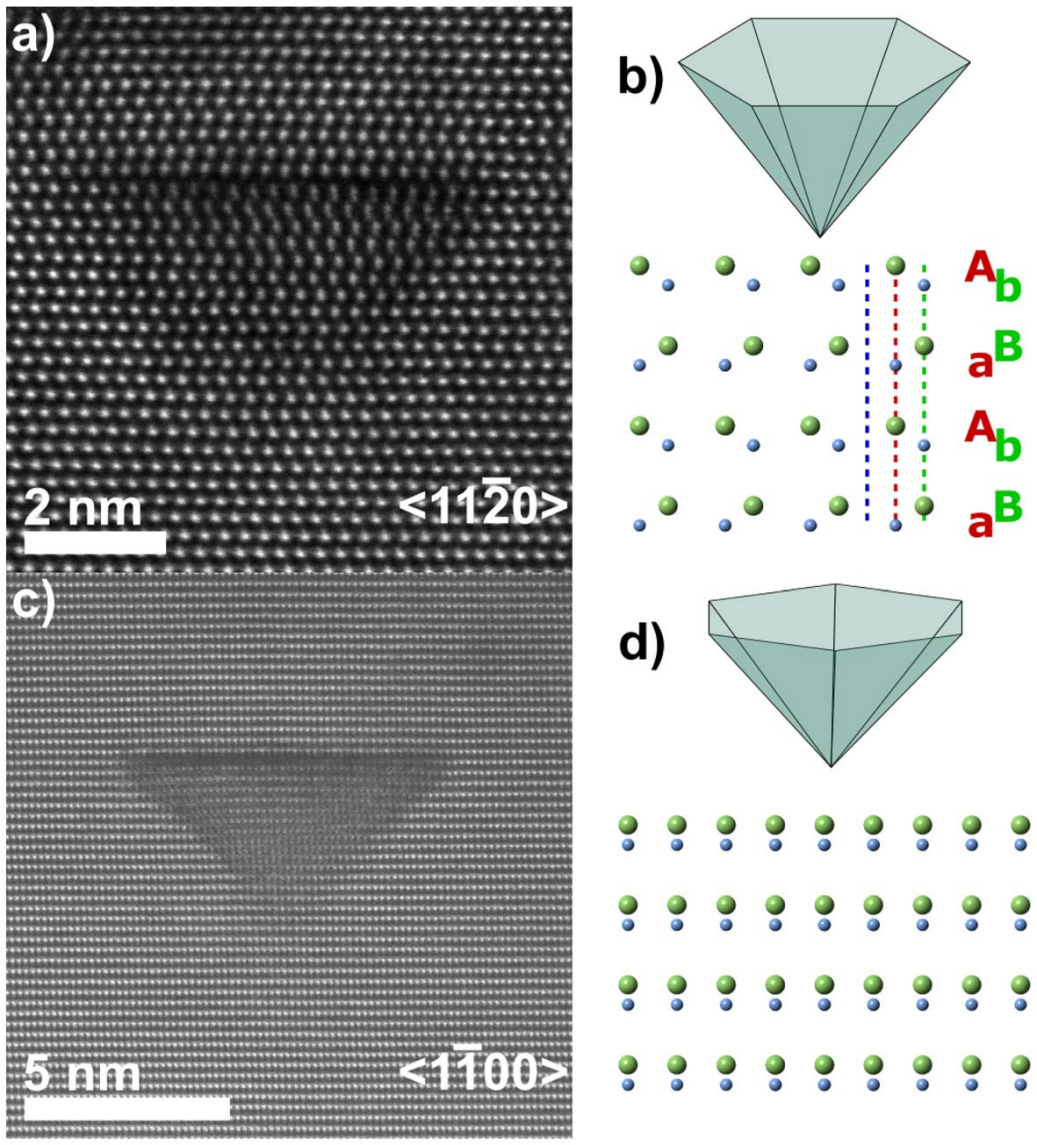
Lattice resolved view of wurtzite (WZ) GaN and the pyramidal defect with a schematic representation of the defect and GaN lattice, viewed along ${<}11\bar{2}0{>}$ in (**a**) and (**b**) and along ${<}1\bar{1}00{>}$ in (**c**) and (**d**). The schematic pyramids are slightly tilted towards the viewer to illustrate the orientation of the hexagonal shape. For the model structures, Ga is light green, and N is light blue.

The previously identified shape and orientation of the hexagonal PID is confirmed by the lattice resolved HAADF-STEM images in Fig. 2a and c, in agreement with previous investigations^18,26,27^. The corresponding schematic structure of Ga-polar GaN, as well as the orientation of the PIDs relative the viewing directions, ${<}11\bar{2}0{>}$ and ${<}1\bar{1}00{>}$, are shown in Fig. 2b and d, which project the defect through a corner and along a facet, respectively. The growth direction ${<}0001{>}$ is up in all the images of Fig. 2 and the letters indicate the stacking order of WZ: *aBbAaBbA*, where uppercase letters indicate Ga and lowercase letters N, and their respective positions either on the A (red) or B (green) position. The hexagonal shape is also confirmed by mapping the intensity-profile of the PIDs by HAADF-STEM (Fig. S1, Supplementary Information online).

**Figure 3:**
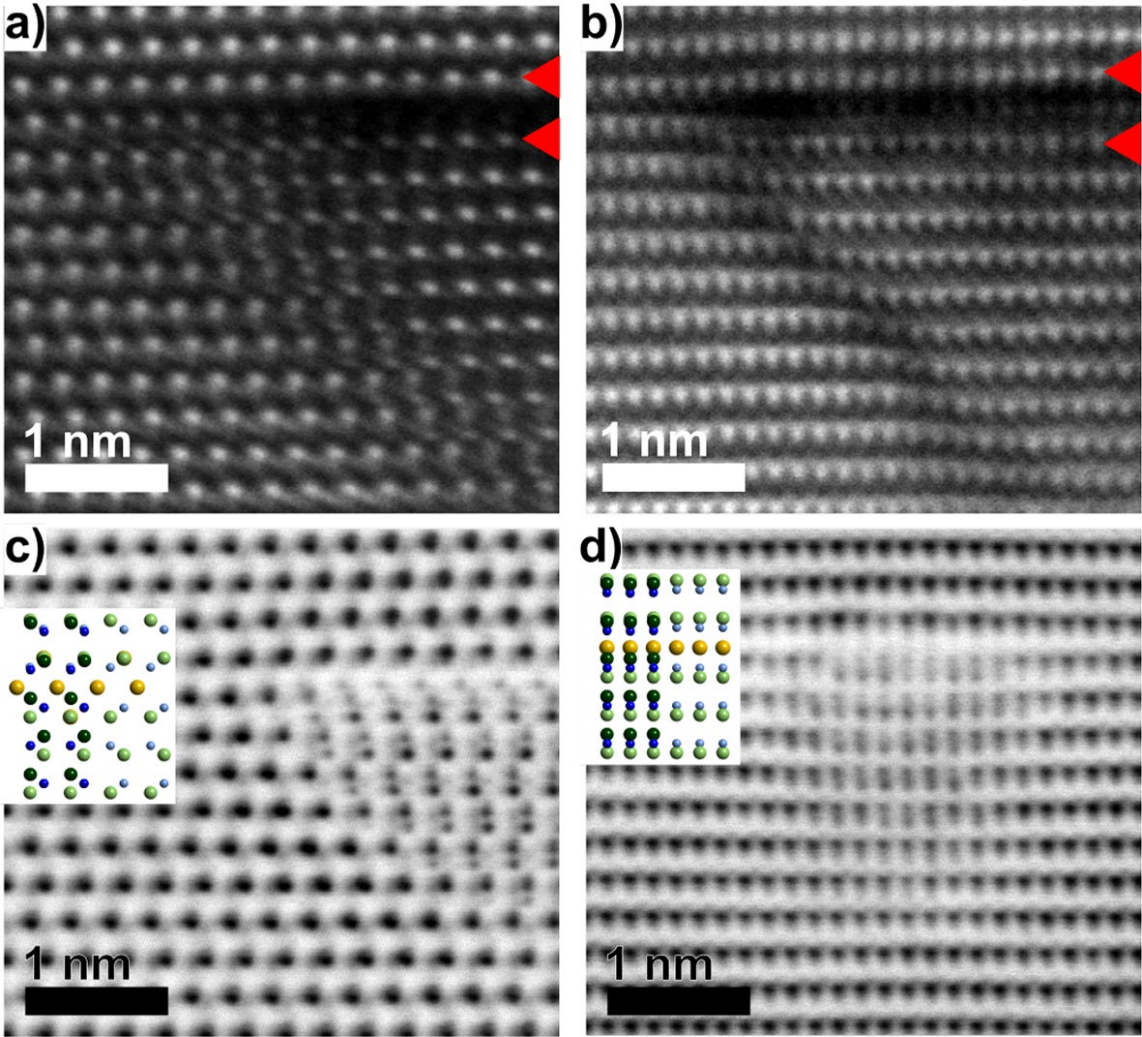
Acquired images of top-left side of the PIDs: by HAADF-STEM (**a**–**b**) and by ABF-STEM (**c**–**d**) for ${<}11\bar{2}0{>}$ a) and (**c**) and ${<}1\bar{1}00{>}$ (**b**) and (**d**) projections respectively. The right side of insets in (**c**) and (**d**) show the top Mg-layer and the inversion, while the left sides show the same but overlapping with the ambient matrix. For the model structures, Ga is light green, N is light blue, and Mg is yellow. The dark green and blue are the corresponding atoms but for the ambient matrix overlapping the structure.

The HAADF-STEM images shown in Fig. 3a,b reveal a reduced atomic column intensity in the top $\{0001\}$ facet. Also, the neighboring layers, indicated by red arrows, exhibit a reduced intensity with additional intensity fluctuations among the individual columns compared to the layers further from the Mg-layer and in the ambient matrix. These observations suggest random substitution by Mg on Ga sites and confirm the findings by Iwata et al.^13^.

The ABF-STEM images in Fig. 3c,d enable visualization of the individual atomic columns (Ga, N and Mg), and hence identification of the polarity. The ambient GaN matrix has a Ga polarity, while inside the pyramids the structure is inversed, i.e. has N-polarity, in agreement with previous findings. The images also clearly reveal that the top Mg-layer occupy *C*-positions (*C*$_{\mathrm {Mg}}$, blue line in Fig. 2b). This establishes the stacking order *AbBaAbC*$_{\mathrm {Mg}}$*aBbAaB*, starting within the PID and along the ${<}0001{>}$ direction, in line with the Northrup model^23^. This stacking order can only be observed in the ${<}11\bar{2}0{>}$ projection and have previously also been suggested from TEM images by Liliental-Weber^28^. Fig. 3c is also shown at higher magnification in Fig. S2, Supplementary Information online, where it is combined with the lines marking the positions in Fig. 2.

All images in Fig. 3 are acquired in thin regions, however, given the projected geometry of the imaging conditions, overlap between PID and ambient matrix is observed. This overlap is schematically demonstrated by the insets in Fig. 3c,d where the inverted GaN below the Mg-layer is overlapped by the ambient matrix (left side of insets).

From Fig. 3b the inclined facets of the PID are clearly visible and reveal a drop in intensity that indicates presence of Mg. The Mg-decorated facet is estimated to include 3 Mg atoms in width, seen in this projection, in agreement with the model described by Romano et al.^22^ for a $\{11\bar{2}3\}$ facet. Also, clearly seen is that the lattice inside the PID appears vertically shifted relative to the ambient matrix where the shift, including the polarity inversion, occurs at the inclined facet.

The shift is measured from Fig. 3, mainly from the Ga atomic layers from HAADF-imaging but also from N and Mg atomic layers in the ABF images. The Ga atoms within the PID are shifted downwards by 1.1 Å and Ga atoms above the PID are shifted upwards by 0.4 Å—both relative to the matrix. These shifts have not been considered in previous models, which motivates a modification of the PID model. Therefore, the models by Northrup^23^ (the top $\{0001\}$ facet) and Romano et al.^22^ (the inclined facets) are here combined with the experimentally measured shifts, into an updated model.

**Figure 4:**
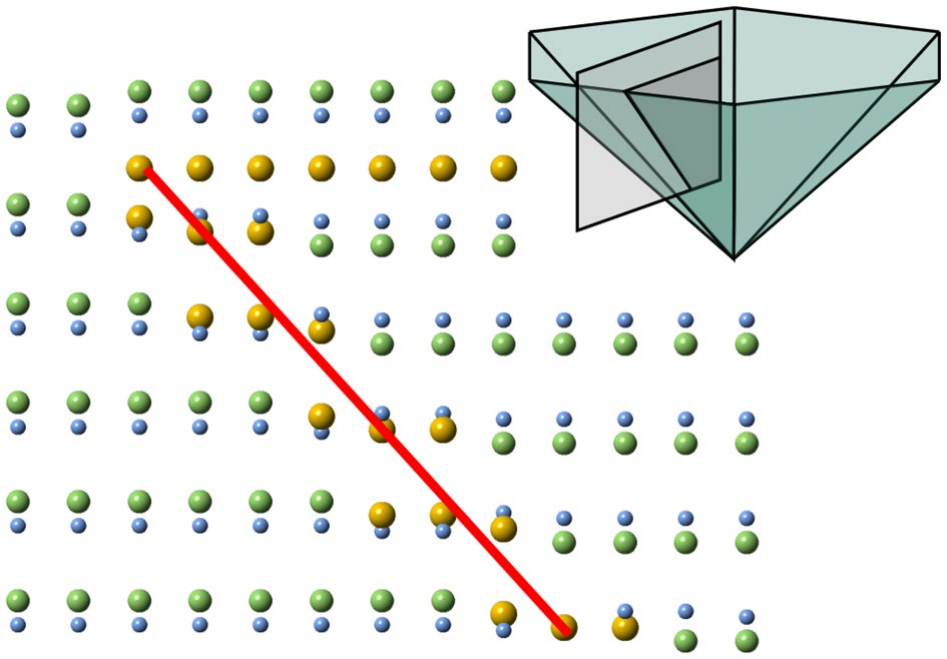
Illustration of the top left corner from the modified combined structure model of the hexagonal PID vs. the ambient matrix along a ${<}1\bar{1}00{>}$ direction. The top-right inset identifies the position of the illustration in the PID. The red line outlines a $\{11\bar{2}3\}$ facet. In the model structure, Ga is light green, N is light blue, and Mg is yellow.

Fig. 4 shows a cross sectional slice of the top left corner of our modified and combined PID model in the ${<}1\bar{1}00{>}$ projection. Inside the PID, the Ga and N atoms are shifted in the ${<}000\bar{1}{>}$ direction, keeping the bond length (Ga-N) of the ambient matrix. At the $\{0001\}$ facet of the model, the position of the Mg layer between adjacent N atoms layers, generates bond lengths (Mg-N) that match the calculated bond lengths of Northrup’s model. Northrup’s calculations show a bond length of 2.33 Å^23^ while a bond length of 2.32 Å is observed in our model. The random substitution of Ga with Mg in the adjacent layers to the flat facet is also included in the model.

The $\{11\bar{2}3\}$ inclined facet is used to describe the present model (as previously described and observed^8,17,21,22^), as this is the facet observed in the current sample (Fig. 3b) and corroborated by recent theoretical calculations^24^. The Mg atoms substitute Ga on three adjacent positions in the layer in agreement with conclusions by Romano et al.^22^. In the model, the Mg atoms in the inversion layer are shifted in the ${<}0001{>}$ direction, creating a transition between the positions of the Ga atoms inside and outside the PID. Following the model by Romano et al., every other layer exhibit two Mg atoms with the bonded N atoms slightly above and one just below, and vice versa. The shift of the Mg atoms corresponds to $\tfrac{1}{3}$ of the total downward shift for Mg atoms positioned above N (ambient matrix vs. PID) and $\tfrac{2}{3}$ of the shift for the Mg atoms positioned below N, to induce a gradual shift corresponding to observations. The associated N atoms are positioned at the corresponding height not occupied by the Mg atom for simplicity, generating a slightly shorter bond length (Mg-N) in this layer compared to the Ga-N bond length in the rest of the structure.

The Mg-atoms in the model structure at the $\{0001\}$ facet are 6-fold coordinated (corresponding to the calculation by Northrup^23^) while those in the inclined inversion layer exhibit 3-fold coordination. This 3-fold coordination is the most likely arrangement considering the inversion of the tetragonal coordination of Ga/Mg moving from matrix into the PID. At the inversion layer, seen in Fig. 3b, the intense atoms shift downwards, suggesting a flattened coordination with the N-atoms, hence a local 3-fold coordination occurs at this layer.

**Figure 5:**
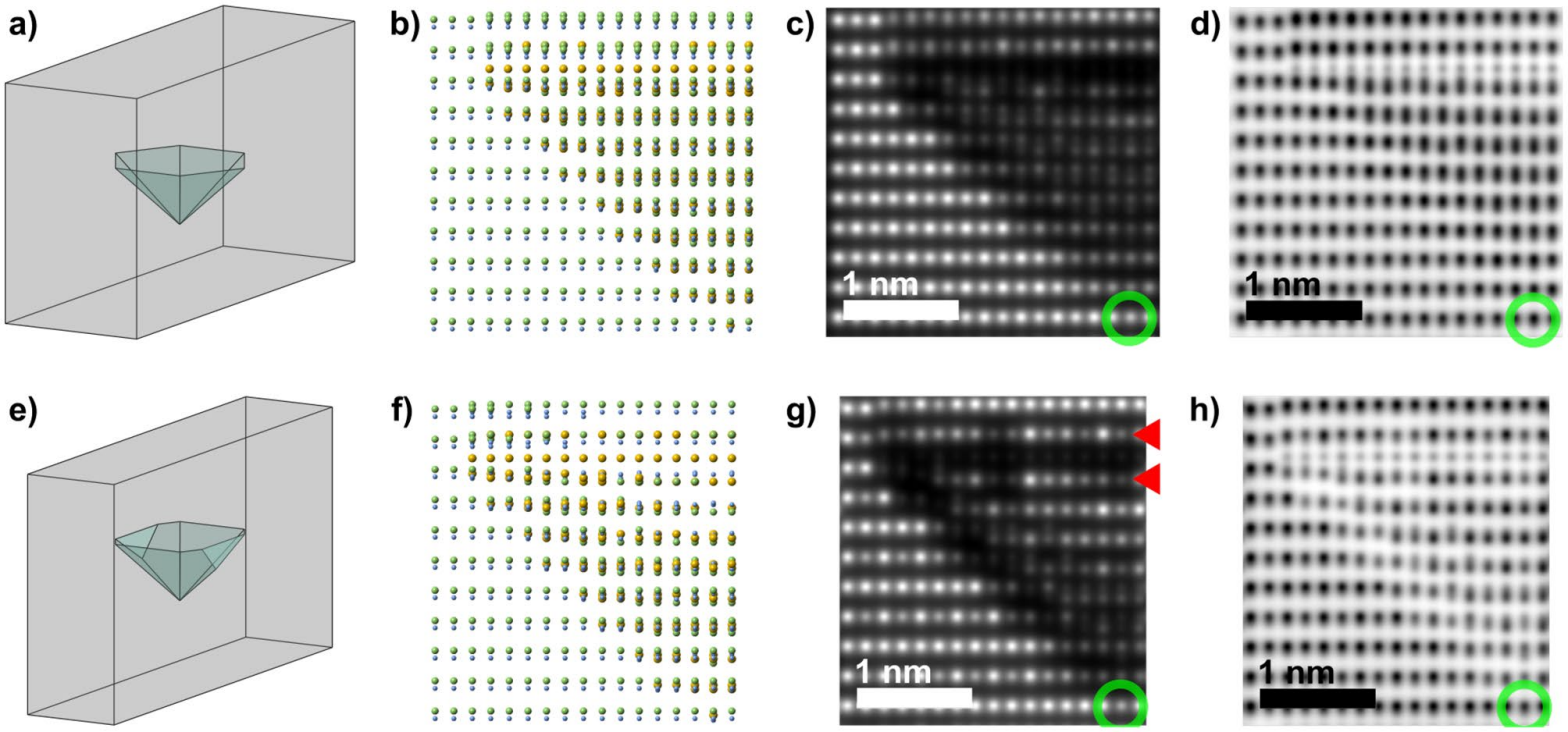
Schematic structure and simulated STEM images of the PID structures in the ${<}1\bar{1}00{>}$ projection for different thicknesses. (**a**) and (**e**) show schematic lamellas of different thicknesses embedding and partly embedding the defect, respectively. (**b**) and (**f**) show the corresponding projected atomic structure model of half the PID, including the apex at the bottom right. (**c**) and (**g**) show the simulated HAADF-STEM images while (**d**) and (**h**) show the corresponding ABF-STEM images for the different thicknesses respectively. The green circles and red arrows identify the apex and the planes adjacent to the Mg-layer, respectively. In the model structure, Ga is light green, N is light blue and Mg is yellow.

Example simulations of the modified PID structure embedded in matrix lamellas with different slab thicknesses are shown for the ${<}1\bar{1}00{>}$ projection in Fig. 5a–d versus e–h (the ${<}11\bar{2}0{>}$ projection is shown in Fig. S3, Supplementary Information online). Schematic drawings of the simulated lamella thickness in relation to the PID are shown in Fig. 5a and e, where Fig. 5a shows a fully embedded PID with continuous overlap between PID and ambient matrix in projection. In Fig. 5e the lamella surfaces truncate the PID, hence the lamella exhibit regions without overlap between matrix and PID. Fig. 5b and f show the projected structures for the respective thickness. Fig. 5c,d and g,h show the corresponding simulated HAADF- and ABF-STEM images. These simulations correspond well with the acquired images in Fig. 3, where the darker Mg-based $\{0001\}$ facet is clearly seen in both Fig. 5c and g. Qualitatively, the intensity of the layers adjacent to the Mg-layer (indicated by red arrows in Figs. 3 and 5) is lower than the ambient matrix, which infers that Mg randomly substitutes for Ga in these layers. Both in the acquired and in simulated images, the atomic columns exhibit intensity fluctuations, supporting the random substitution of Ga with Mg within these layers. Within Fig. 5g the fluctuation is significant, deriving from the thin simulated structure (less than 7 nm), in which single atoms in projection relatively affect the contrast more strongly.

The experimentally observed lattice shift within the PID is reproduced in the simulated images, supporting the modified model. At the inclined interfaces, the intensity reduction originates from the presence of Mg. However, the thickness of the sample affects the visibility of the inclined facets substantially, compared to the top $\{0001\}$ facet. While the simulation in Fig. 5g shows the near ideal case, the region near the apex still projects a significant overlap with the embedding matrix. Image contrast and lattice shift become blurred and are not visible near the apex, even for the thinnest sample. This is also reflected in Fig. 3b, where the inclined facets compare well with the simulations in terms of shift and intensity loss. Overall, the simulated and acquired images match qualitatively, and verify the adapted model.

**Figure 6:**
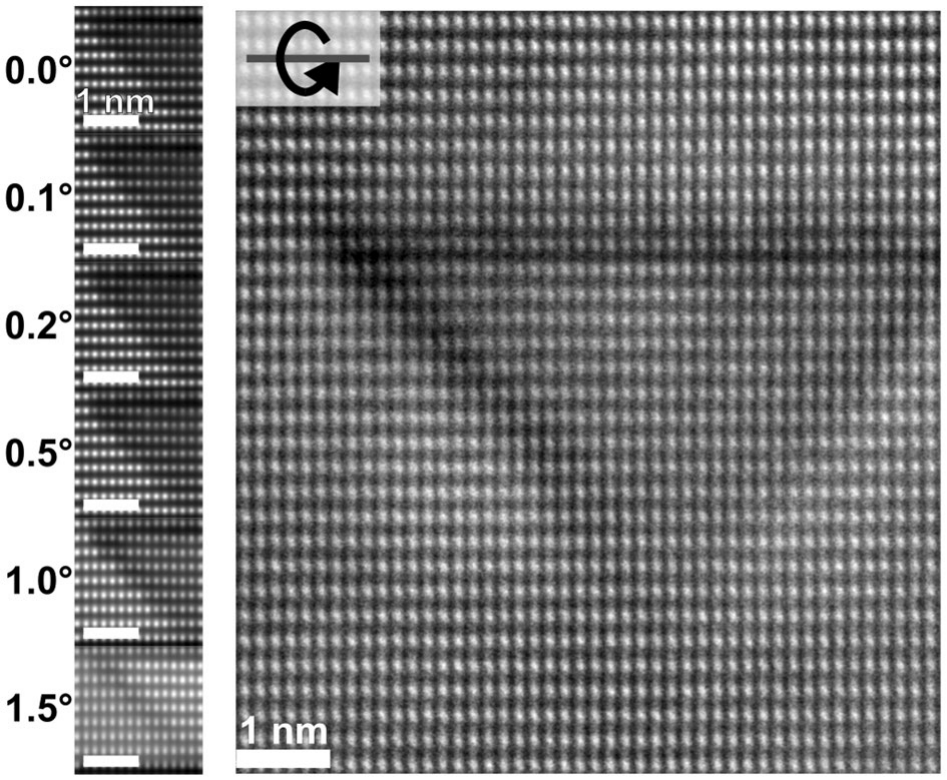
Comparison of acquired HAADF-STEM image (right) to simulated projections with overlap between matrix and PID. The simulated PID has the same Mg-thickness as the model in Fig. 4. The simulations show increasing rotation around the horizontal axis.

It is also found that the image contrast, associated with the defect, strongly depends on sample tilt. Fig. 6 shows a PID where dark regions clearly outline the shape. Image simulations of the improved model match the dark outline well if a tilt around a horizontal axis in the structure’s projection is considered. The exact tilt angle is not attempted to be matched, but the fact that the contrast appears when tilting the simulation confirms the assumption of tilt in the acquired image. Accordingly, the acquired image in Fig. 6 is a fingerprint that further support the three Mg-atoms wide inversion layer outlining the pyramid’s $\{11\bar{2}3\}$ facets. For simplicity, the simulated structure had full occupancy of Mg instead of the predicted $\tfrac{3}{4}$^22^, but this difference is only expected to slightly change the contrast of the simulated interfaces compared to the real one. The contrast is believed to arise from smearing in the vertical direction due to tilt, reducing the effect of slightly displaced atoms (strain around the interfaces) in relation to the projected matrix, leaving the Z-contrast more pronounced. The intensity drop in Fig. 3b also indicate a change in projected atomic number at these inclined inversion layers but Fig. 6 highlights this clearer and for the entire outline of the PID. This suggests that the same composition and structure exists at all inclined facets. By extension, the structure used to describe the inclined facets, can be extended towards the apex.

**Figure 7:**
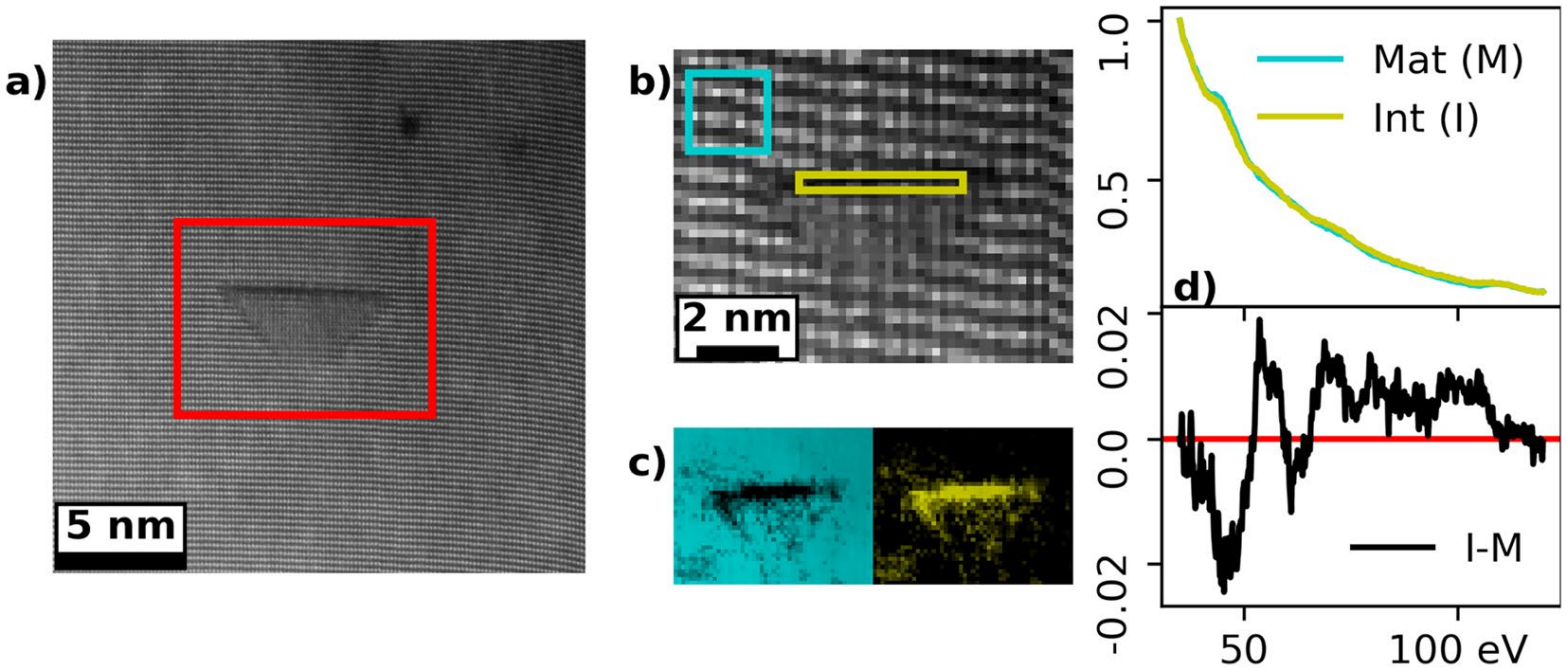
STEM-EELS mapping by MLLS of a PID observed in the ${<}1\bar{1}00{>}$ direction. (**a**) shows the HADF-STEM overview image of the analyzed PID, with a red rectangle marking the analyzed region. (**b**) is the simultaneously acquired HAADF-STEM image of the spectrum image with the MLLS reference regions (blue matrix and yellow Mg rich) marked. (**c**) shows the maps for matrix and Mg rich regions in their corresponding colors. (**d**) shows the respective intensity normalized components in their corresponding colors, from 35 to 120 eV. The difference between these two (I﻿−M) is shown in the lower panel.

Previous studies have confirmed Mg to be present at the pyramid’s $\{0001\}$ facet, however the present result indicate that Mg is also present at the inclined facets. To verify this, EELS spectrum imaging (SI) of the PIDs was performed, and the distribution of Mg was mapped using the Mg-L$_\mathrm {2,3}$ energy-loss edge ($\sim$51 eV). Since the edge is near the low loss region, the Mg-edge is superimposed on other signals, e.g. the GaN bulk plasmon. Thus, to differentiate between the Mg-edge and other signals, multiple linear least square fit (MLLS) was employed, and the results are shown in Fig. 7. Fig. 7a shows the ${<}1\bar{1}00{>}$ projection of a PID, with the area used for spectrum imaging highlighted. The simultaneously recorded HAADF-STEM signal for the SI is shown in Fig. 7b where two reference regions are marked with colored rectangles corresponding to the matrix (Mg-poor) and the pyramid’s $\{0001\}$ facet (Mg-rich). Their respective MLLS maps are shown in Fig. 7c. From the maps it is evident that the Mg rich signal is not only present at the top $\{0001\}$ facet of the PID, but also present at the inclined facets. The Mg signal diminishes towards the apex of the PID, associated with the projected thickness of the defect, and hence the number of Mg atoms. Also, the signal is less pronounced compared to the $\{0001\}$ facet. This is expected since the occupation is proposed by Romano et al. to be $\tfrac{3}{4}$ Mg and $\tfrac{1}{4}$ Ga in the inversion layer^22^. The diminishing Mg intensity in Fig. 7c, points to a homogeneous inclined inversion layer, also close to the apex.

A comparison of the two components is shown in Fig. 7d, with the individual components at the top and their difference (I−M) in the lower panel, where the difference spectrum corresponds well with the Mg-L$_\mathrm {2,3}$ edge^29^. The plasmon signal of GaN and the signal from the Mg-L$_\mathrm {2,3}$ edge (from a MgO sample) are compared in Fig. S4, Supplementary Information online. Accordingly, the mapped signal is that of Mg and no artefact.

The Mg concentration measured by SIMS is $6.70 \times 10^{19}$ $\mathrm {cm^{-3}}$. However, C–V measurements showed a $N_A - N_D$ equal to $4.90 \times 10^{18}$ $\mathrm {cm^{-3}}$, indicating that a large concentration, around $6.2 \times 10^{19}$ $\mathrm {cm^{-3}}$, of the Mg atoms are electrically inactive. Density and size of multiple (> 100) PIDs were estimated by STEM (illustration on the measurements are shown in Fig. S5, Supplementary Information online) from several sites for improved statistics. The top region of the GaN:Mg layer was analyzed in order to stay away from the uneven distribution and sizes closer to the nucleation layer (Fig. 1). The average top area of the PID varies depending on the specific site but falls in the range 22.9 to 32.7 nm$^\mathrm {2}$. Using the same method as Narita et al.^30^ for the top $\{0001\}$ facet, but also adding the Mg found in the inclined inversion region according to the model confirmed above (Fig. 4), the concentration of the Mg located in the PIDs is $5.9\pm 1.2\times {10}^\mathrm {19}$ $\mathrm {cm^{-3}}$.

## Discussion

Previously, Mg has not, to our knowledge, been directly verified at the inclined facets of the hexagonal PID^7,18,31^, however, the present EELS analysis together with contrast variations observed by STEM, verify that the inversion domains at the inclined facets contains Mg and are well defined. The model presented here is derived from Romano et al.^22^ as well as Northrup^23^ with refined distances from the acquired high-resolution STEM images. The observations are in line with earlier TEM studies of the PIDs, e.g. in terms of the inclined facets as discussed by Lilliental-Weber^28^, with improved details from ABF-STEM and direct confirmation of the Mg-distribution within the inclined facets by EELS.

As this study focuses on properties and composition of the inclined facets of the pyramidal defects it also raises questions about its apex. It is inherently the most interesting part of the PID as it is what causes the defects to form in the first place (for Ga-polar GaN). Insights into its properties could help prevent them to form. The shape of the defect also makes this part the most difficult to analyze. It will always be embedded in a relatively thick ambient matrix and its potential compositional signal is the lowest possible. With similar spectroscopic signals of Ga and Mg it is difficult to directly determine Mg as being part of the apex, especially also considering that the structure is sensitive to the electron beam (Fig. S6, Supplementary Information online). The structure imaged and mapped here indicates that the inclined facets are homogeneous and originate from a very narrow tip. This suggests that the apex consists of a similar composition of atoms, which are arranged in a similar manner as in the inclined facets. In other words, for high Mg concentrations Mg is randomly incorporated with 3-fold coordination, instead of substituting Ga, causing the defect to form. Then this coordination promotes formation of inclines, essentially spreading from one point when growing until terminated by a $\{0001\}$ Mg layer, forming a pyramid.

From the images in Fig. 3 the lattice within the PID is slightly distorted, potentially due to strain from the arrangement at the inclined facets. Correspondingly, after a certain number of layers the relative vertical shift of the Ga-atoms to the ambient matrix increases, creating the shift (ambient matrix to inside the PID) observed in Fig. 3. Potentially, this relaxation is due to increased lateral size of the PID further from the apex. This would mean that at a certain point, enough shift is generated allowing for the full Mg-layer to form, with the energetically favorable distances as calculated by Northrup. This terminates the PID, and the defect is completed.

The amount of Mg bound to PIDs ($5.9\pm 1.2\times {10}^\mathrm {19}$ $\mathrm {cm^{-3}}$) could account for the concentration of electrically inactive Mg ($6.2 \times 10^{19}$ $\mathrm {cm^{-3}}$) measured in this sample^14^. This would imply that the formation of PIDs is the main limiting factor of *p*-conductivity when using Mg concentrations in the low-to-mid $\mathrm {10^{19}}$ $\mathrm {cm^{-3}}$ and above. However, it should be noted that the difference between the H-passivated Mg acceptors before annealing of $1.4 \times 10^{19}$ $\mathrm {cm^{-3}}$ (assuming all the available H is bound to Mg) is larger than the net acceptor concentration $N_A - N_D$ after annealing ($4.90 \times 10^{18}$ $\mathrm {cm^{-3}}$). This suggests the presence of compensating donors, most probably nitrogen vacancies, V$_\mathrm {N}$, and Mg-V$_\mathrm {N}$ complexes, the density of which depend on the MOCVD growth conditions^14^.

We have presented a detailed study and analysis of the hexagonal pyramid-shaped polarity inversion domains formed during growth of Mg-doped GaN using high-resolution STEM-EELS. Here we have employed sensitive HAADF- and ABF-STEM imaging for improved resolution of the structural arrangement of the PIDs. These methods have enabled us to establish an improved comprehensive model of the PID structure in relation to the ambient matrix. Furthermore, the results unambiguously show that Mg is present at all interfaces between PID and matrix. The spectroscopy and imaging in combination with image simulations of the model confirm that the inclined facets have a well-defined inversion layer containing Mg atoms, according to the adjusted model with a 3-fold coordination. We find that the estimated total amount of Mg in the PIDs accounts for the amount of electrically inactive Mg after growth for the sample under investigation. The established model allows for more accurate evaluation of Mg segregated at the PID, necessary for understanding the main limiting factor for *p*-type conductivity in GaN against alternative compensating donor or passivation sources.

## Methods

Intentionally *p*-doped (Mg) GaN (GaN:Mg) $\sim$600 nm thick was grown in a hot-wall metalorganic chemical vapor deposition (MOCVD) reactor on top of a silicon carbide (4H-SiC) substrate and a 75 nm thick aluminum nitride (AlN) nucleation layer. The doping resulted in a Mg concentration of $6.70 \times 10^{19}$ $\mathrm {cm^{-3}}$ confirmed by secondary ion mass spectrometry (SIMS). The sample was part of a larger study for investigating different doping concentrations under various growth conditions and the related structural and electrical properties, which is presented elsewhere^14^.

SIMS was measured on the sample for a quantitative depth profile of Mg dopant and background impurity elements (H, C, Si and O), where the thicknesses of GaN:Mg and AlN layers are confirmed as well. The SIMS measurement was performed by EAG Labs^32^ and the average atomic concentrations were estimated using the SIMSview program from the same company. C-V measurements, quantifying the net acceptor concentration ($N_A - N_D$) were performed in a Hg-probe setup with a 4284A LCR meter from Agilent (series-mode measurements in the frequency range: 1–10 kHz).

Conventional cross-sectional TEM sample preparation was performed using mechanical cutting, mounting in Ti-grids followed by mechanical polishing and final Ar-ion thinning at 5$^\circ$ using a Gatan PIPS Model 691 to reach electron transparency. Samples were prepared to enable observation of the GaN-structure in both ${<}11\bar{2}0{>}$ and ${<}1\bar{1}00{>}$ directions by cutting two pieces 90$^\circ$ from each other and mounting both in the Ti-grid. To ensure high quality regions with little amorphous material on the surfaces, the TEM-samples were finally polished using a Gentle Mill system (Technoorg Linda Ltd.) using low energy Ar-ions (< 1 keV) for $\sim$5 minutes on each side at 10 and 15$^\circ$ incidence, with full rotation^33^.

For precise measurement of thickness of the sample, a reference lamella was prepared with a viewing direction of ${<}1\bar{1}00{>}$ using a focused ion-beam system (FIB, FEI Helios NanoLAB 600). Ga-ions at 30 kV and currents ranging between 9.3 nA and 98 pA were used. Also, a final polishing step was performed at 2 kV and 28 pA. The cross-sectional lift-out was performed onto a TEM Cu-grid and the lamella was thinned to $\sim$100 nm.

Transmission electron microscopy was performed using the Linköping double-corrected FEI Titan$^\mathrm {3}$ 60-300, operated at 300 keV. HAADF imaging was performed using collection semi-angles between $\sim$66 and 200 mrad. ADF imaging at angles down to $\sim$21 mrad was used to enhance the image contrast contribution from strain. ABF imaging, with collection semi-angles between $\sim$4 and 43 mrad, was used to enhance image contrast for Mg and N relative to Ga, which dominates the HAADF and ADF image contrast. Atomically resolved images were acquired both as single scan frames as well as reconstructed series using the *Smart Align*^34^ plugin for *Digital Micrograph* (Gatan Inc.).

EELS was acquired using a Gatan GIF Quantum detector, with a convergence semi-angle of 22.0 mrad and a collection semi-angle of 56.5 mrad. The spectrometer was used in dual-EELS mode with a dispersion of 0.1 eV/channel and at ranges -20.0–184.8 and 28.0–232.8 eV (exposure times 0.5 ms and 49.5 ms respectively). Multiple linear least square (MLLS) fitting of regions around the PID was conducted using a Python script (provided as Supplementary Information online) using the *Hyperspy*^35^ library for Python. The fitting was performed by averaging spectra from two regions, one of the ambient GaN matrix, and one of the known Mg-containing flat $\{0001\}$ facet. The respective components were fitted and subsequently mapped across the image. For the thickness measurements the EELS detector was used in single spectrum mode and at: 0.25 eV/channel, range: -50.0–462.0 eV, exposure time 0.2 ms, and same convergence and collection semi-angles as above. The mean-free-path was calculated using the built-in function of the Gatan *Digital Micrograph* software and to correlate this to the actual thickness the same settings were used on the reference FIB lamella with known thickness of the same sample and observation direction.

The PID and ambient matrix were simulated and visualized using *CrystalMaker 9.2* and exported as a CIF-file. Fine-tuning of the parameters such as size, shape, shifts, atomic distances and replacement of atoms was done with a Python script which edited the CIF-file. Simulated STEM images of the CIF-file were produced using *Dr. Probe*^36^. Input parameters for the simulations matched those of the microscope (in a corrected state) and collection angles for the simulated images were: ABF: 12–24 mrad, ADF: 40–150 mrad, and HAADF: 80–200 mrad (applied source size: 0.35 Å).

## Supplementary Information

Supplementary Information.

## Data Availability

All data generated or analysed during this study are included in this published article (and its Supplementary Information files).
