# Supplementary material for: Mg segregation at inclined facets of pyramidal inversion domains in GaN:Mg
Source: Sci Rep. 2022 Oct 26;12:17987. doi: 10.1038/s41598-022-22622-1 (PMC9606308; doi:10.1038/s41598-022-22622-1)
Supplement: Supplementary file 1 — Supplementary Information. [file 41598_2022_22622_MOESM1_ESM.pdf]

Supplementary material – Mg segregation at inclined facets of pyramidal inversion domains in GaN:Mg

Axel R. Persson, Alexis Papamichail, Vanya Darakchieva, Per O. Å. Persson

1 Shape and polarity of the PIDs

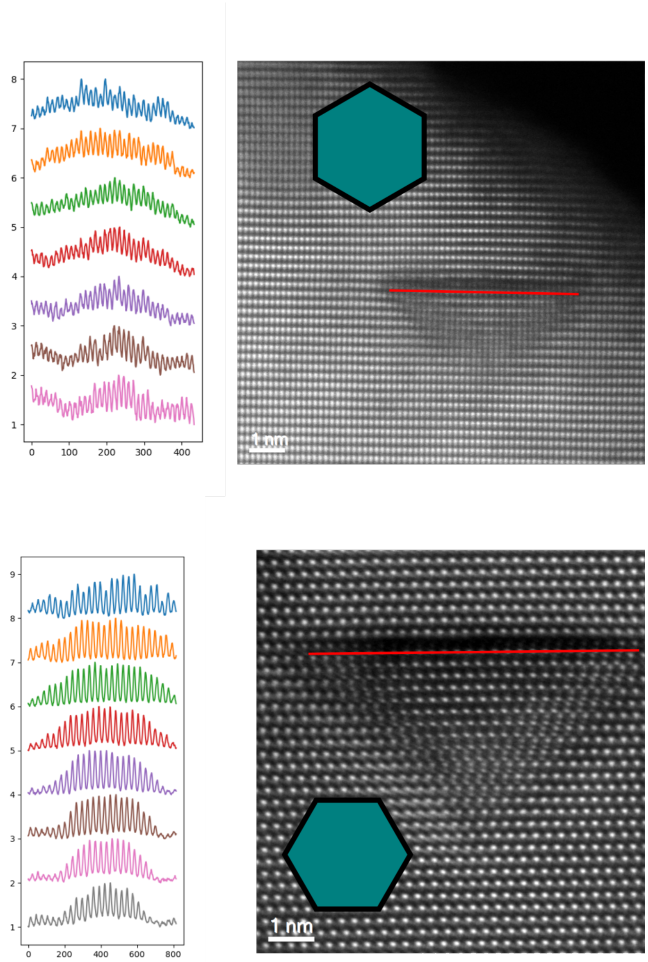

Figure S1: Horizontal line profile of atomic columns belonging to pyramids. Top example shows the $<1\bar{1}00>$ projection while the bottom shows $<11\bar{2}0>$. The acquired HAADF images are shown and the top profile of each pyramid are marked with a red line. This line shows the extent of the profile. Profiles are normalized between 0 and 1 and shifted vertically for comparison. Insets for each pyramid shows the projected direction of the pyramid shape and the plotted profiles agrees with the thickness being a pointed corner for the $<1\bar{1}00>$ projection and semi-flat middle section for the $<11\bar{2}0>$ projection. Note the uneven intensity in the top-layer (blue) for both projections. This is due to the random replacement of Ga with Mg.

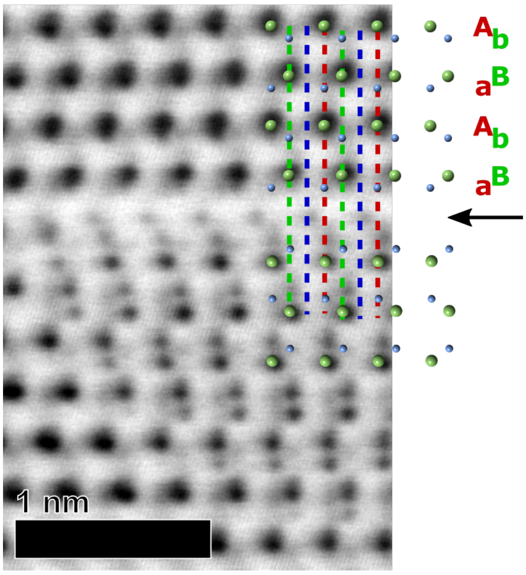

Figure S2: High magnification ABF-STEM image of the top of one pyramid. The lattice is overlayed with the structure of WZ and lines highlight the lateral positions in the ABABAB stacking. However, the top layer of Mg (arrow) positions itself on the blue lines, the C-position. Also, the structure within the pyramid is inverted, following ABABAB stacking. Here it is seen with some overlap from the ambient matrix.

2 Simulation of lamella thickness <11$\bar{2}$0>

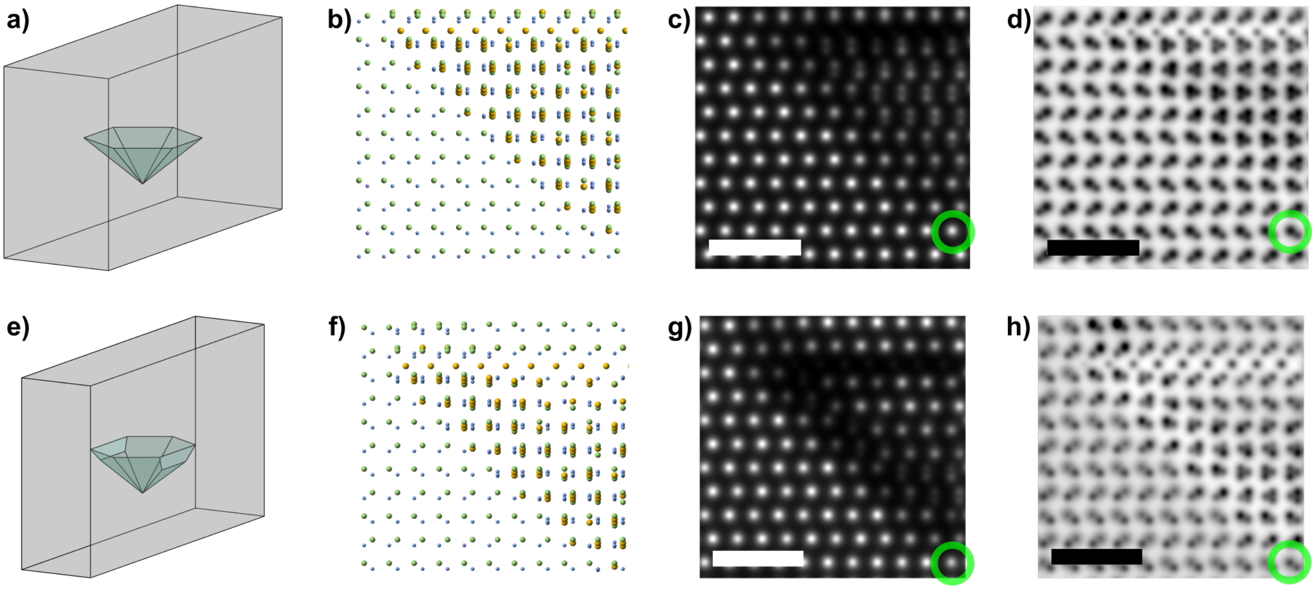

Figure S3: Schematic and simulated cases of TEM imaging of the pyramids in the <11$\bar{2}$0> projection. a) and e) show lamellas projected in the TEM of different thicknesses, where a) has matrix overlapping the whole pyramid in projection (slightly thicker than the pyramid) and e) clips the pyramid, creating a region that has only pyramid domain in projection (slightly thinner than the pyramid). b) and f) show the cases respectively in a simulated atomic model of half the pyramid, including the apex at the bottom right. Colors in the atomic model: green – Ga, blue – N, yellow – Mg. c)-d) simulate STEM images for the first case in HAADF and ABF modes respectively, and g)-h) do the same for the second case (green circles mark the apex). Scalebars in the bottom left of the simulated images are all 1 nm.

3 EELS

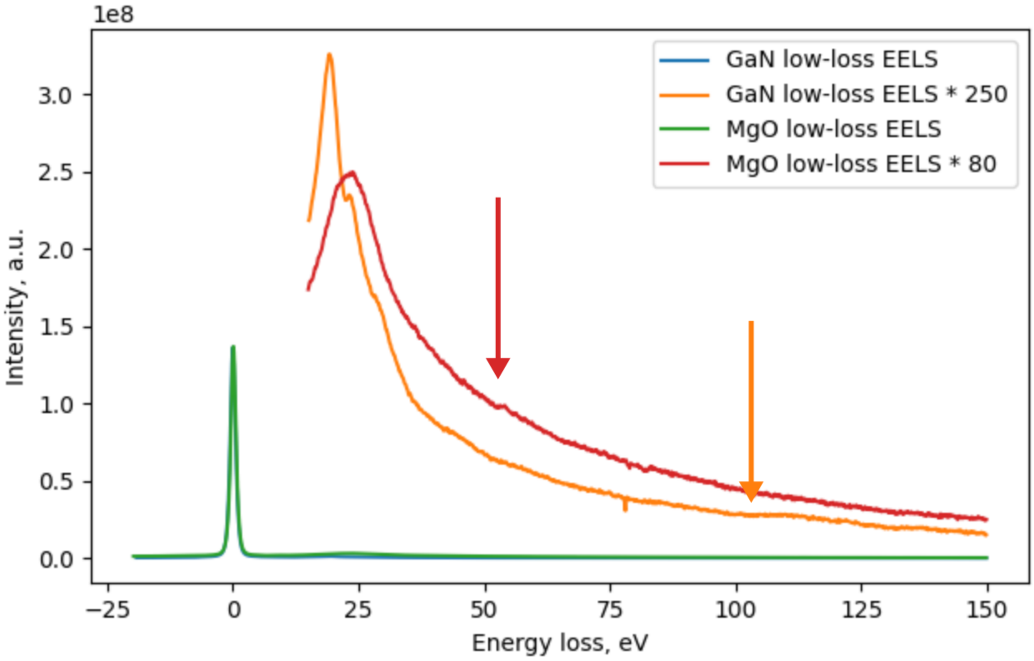

Figure S4: Illustration of the plasmon peaks for the ambient matrix GaN compared to MgO. They have different intensity but are here matched to be compared. The plasmon-regions and up (> 15 eV) are multiplied with different factors for comparison. As can be seen for GaN, there are multiple plasmon peaks/features as high as 50 eV with diminishing intensity. From the MgO signal we see the Mg–$L_{2,3}$ just above 50 eV (edge marked by an arrow with corresponding color). This illustrates the potential overlap and motivates the use of MLLS as described in the main text, as the background for Mg (∼51 eV) can not be properly subtracted. The minor edge of Ga with a delayed onset at ∼103 eV is faintly seen here (also marked by an arrow with corresponding color), but not included in the MLLS analysis in the main text.

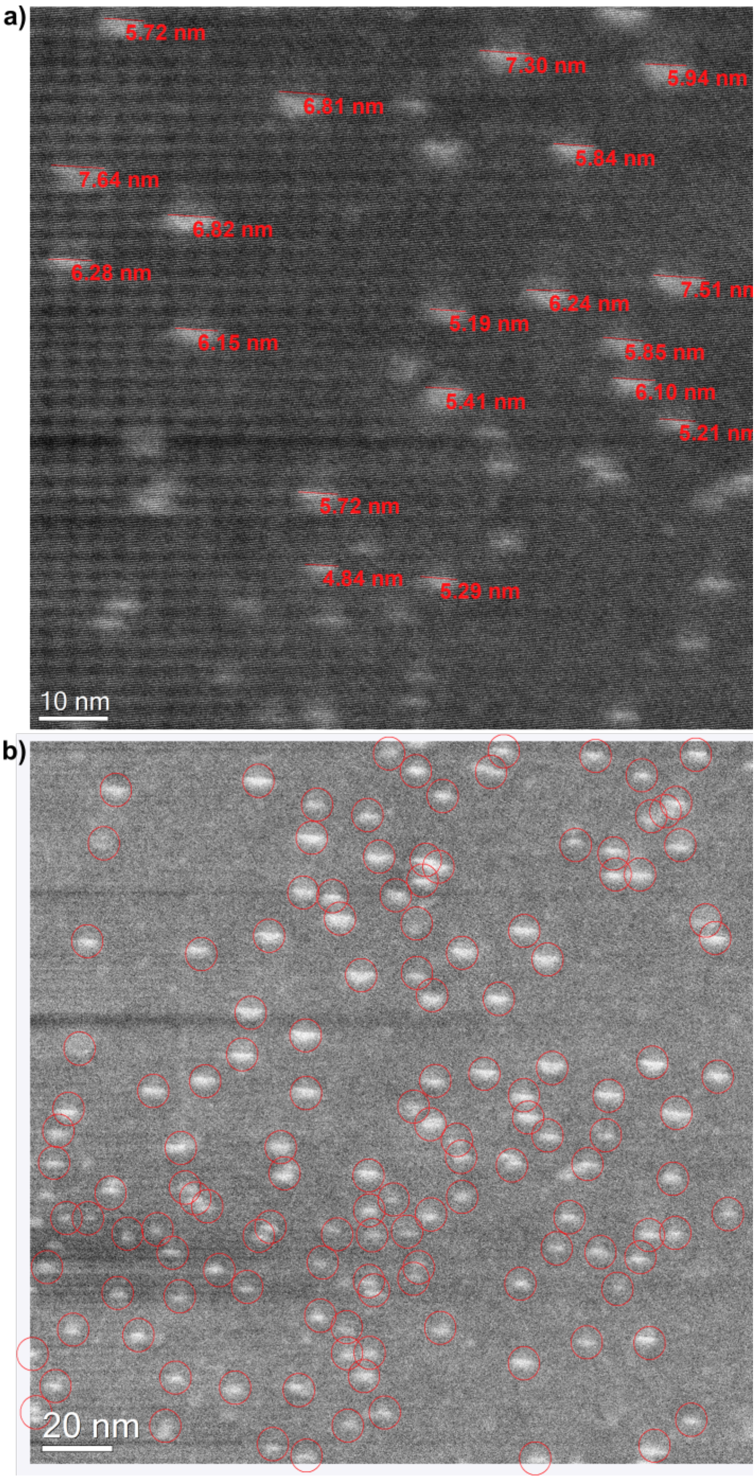

Figure S5: Illustration of size and concentration measurement of PIDs from one of the sites. a) shows a higher magnification making measurements of PIDs with clear width in <1$\bar{1}$00> projection possible. This is used to estimate an average size in the specific region. b) shows manual counting of the PIDs in an image. Since the width and height of the image is known and the thickness is measured through mean-free-path the concentration PIDs can be estimated. The range of Mg concentration presented arises from multiple sites measured for improved statistics and some variation of size are present. The method of manually counting defects in a region also accounts for some margin of error.

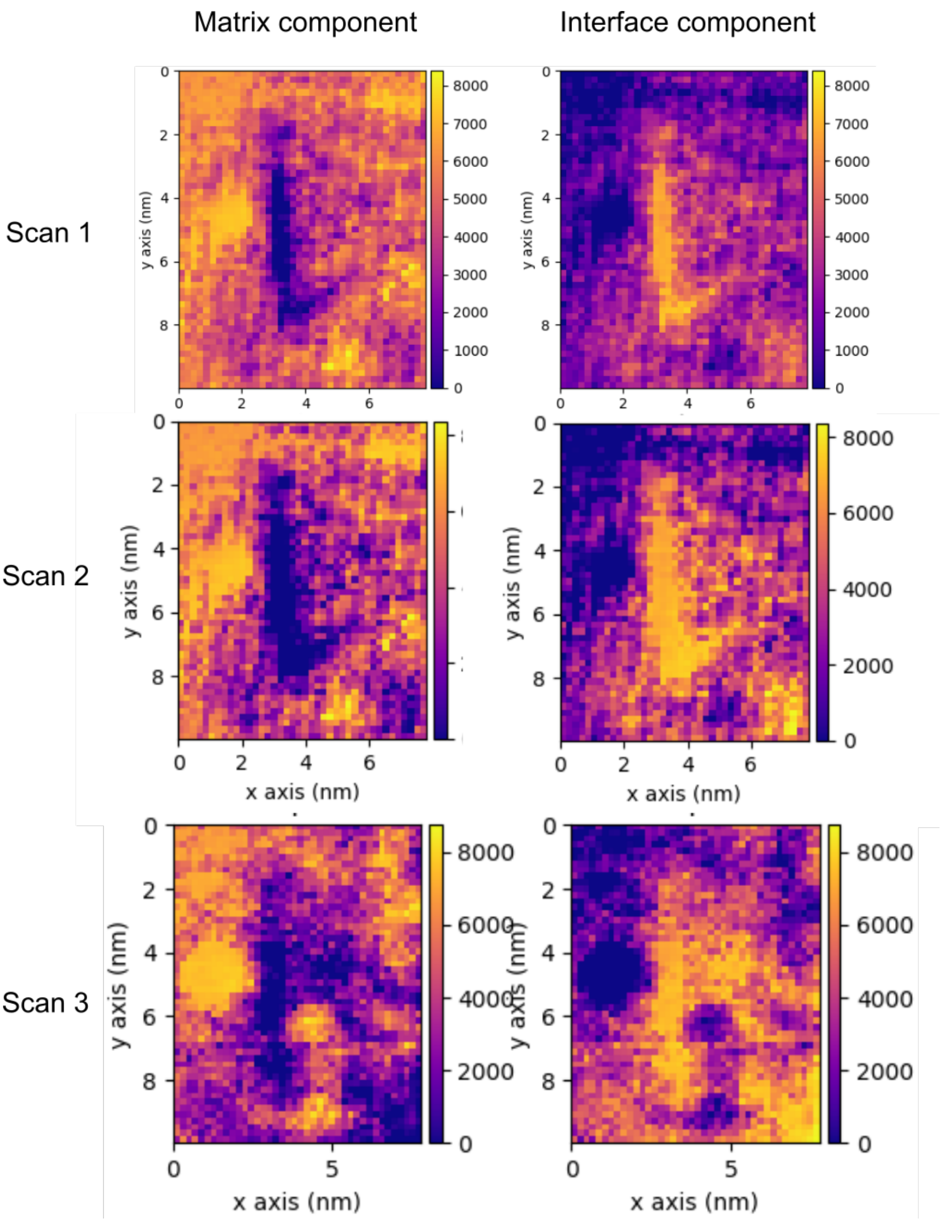

Figure S6: MLLS EELS mapping of two components, just as in the example shown in the main text (figure 4). The components are: the ambient matrix and the interface, shown in left and right columns respectively. The three scans are made using the same settings described in the main text and made just after each other, in series. The results clearly show a degradation of the Mg-interface structure, indicate electron-beam induced damage.

4 MLLS Python script

Import the needed packages

Below follows a workflow to create the figure of MLLS fitting. The data is loaded, fitted and displayed, with comments throughout.

In [1]:

```
#Import packages
#%matplotlib qt #comment for in-line figures and uncomment for separate windows
import hyperspy.api as hs
import matplotlib.pyplot as plt
import scipy.misc
import numpy as np
from scipy import ndimage
import matplotlib.font_manager as fm
import matplotlib.patches as patches
from mpl_toolkits.axes_grid1.anchored_artists import AnchoredSizeBar
import matplotlib.gridspec as gridspec
import warnings
warnings.filterwarnings("ignore")
```

```
WARNING:hyperspy_gui_traitsui:The module://matplotlib_inline.backend_inline matplotlib backend is not compatible with the traitsui
GUI elements. For more information, read http://hyperspy.readthedocs.io/en/stable/user_guide/getting_started.html#possible-warning
s-when-importing-hyperspy.
WARNING:hyperspy_gui_traitsui:The traitsui GUI elements are not available.
```

Load and treat the data

Select which scan to use and set parameters. Then load the data (dm3) using Hyperspy.

In [2]:

```
#setup settings
site_name = '9'
nbr_of_components = 2
E_range = [35.,120.]
max_ZLP_pos = 10.
```

In [3]:

```
#Load the files: DF, survey, HL, LL
hl = hs.load(site_name + '/EELS Spectrum Image (high-loss) (dark ref corrected).dm3')
ll = hs.load(site_name + '/EELS Spectrum Image (low-loss) (dark ref corrected).dm3')
df  = hs.load(site_name + '/DF.dm3')
survey = hs.load(site_name + '/SI Survey Image.dm3')
```

Align the SI data according to the low-loss (ll) zero-loss peak

In [4]:

```
#Align the acquired EEL spectra in every pixel to the zero-loss peak (3 times for improved accuracy)
for k in range(3):
    ll.align_zero_loss_peak(subpixel=True, also_align=[hl], signal_range=(ll.axes_manager['Energy loss'].offset,max_ZLP_pos),show_
```

```
Initial ZLP position statistics
-------------------------------
Summary statistics
------------------
mean:   1.94
std:    0.0999

min:    1.7
Q1:     1.9
median: 1.9
Q3:     2
max:    2.2

Initial ZLP position statistics
-------------------------------
Summary statistics
------------------
mean:   8.5e-16
std:    0.0487

min:    -0.0389
Q1:     -0.0389
median: -0.0389
Q3:     0.0611
max:    0.0611

Initial ZLP position statistics
-------------------------------
Summary statistics
------------------
mean:   0
std:    0

min:    0
Q1:     0
median: 0
```

```
Q3:      0
max:     0
```

Define the two regions of interest (ROI) used as components. Add these two spectra to "component_list". The positions are manually selected as two rectangles.

In [5]:

```
#Extract components scriptwise (not interactively)
roi_list = []
component_list = []
legend_list = []
image_list = []

roi_list.append((39,49,3,13))
roi_list.append((14,35,16,18))#Location (indices) selected manually

for k in range(nbr_of_components):
    curr_component = hl.inav[roi_list[k][2]:roi_list[k][3],roi_list[k][0]:roi_list[k][1]].sum().squeeze()
    max_val = curr_component.isig[E_range[0]:E_range[1]].max(axis=0).data[0]
    curr_component = curr_component/((roi_list[k][3]-roi_list[k][2]+1)*(roi_list[k][1]-roi_list[k][0]+1))
    component_list.append(curr_component)
    legend_list.append('Component ' + str(k))
```

Perform the multiple least linear square (MLLS) fitting using the defined components ("component_list").

First the components are added to the model ("m") and then the fitting is performed ("m.multifit()"), then the resulting fit of each component is saved to "image_list"

In [6]:

```
#Perform MLLS
m = hl.create_model(auto_background=False)
for k in range(nbr_of_components):
    curr_model = hs.model.components1D.ScalableFixedPattern(component_list[k])
    curr_model.shift.free = False
    curr_model.xscale.free = False
    curr_model.yscale.bmin = 0
    m.append(curr_model)
    m[k].name = 'Component ' + str(k)
m.set_signal_range(E_range[0],E_range[1])
m.multifit(bounded=True, iterpath='flyback')
for k in range(nbr_of_components):
    image_list.append(m[k].yscale.as_signal().T)
```

Presentation of data

Collect the data into presentable figures.

The fitted maps of "image_list" are displayed, followed by the two components plotted as spectra (including their difference). Lastly the image of the region is shown with rectangles marking the positions of the ROIs.

In [7]:

```
#Create display of the images
max_vals = []
min_vals = []
for k in range(nbr_of_components):
    max_vals.append(image_list[k].max().data[0])
    min_vals.append(image_list[k].min().data[0])
max_val = max(max_vals)
min_val = min(min_vals)
hs.plot.plot_images(image_list, tight_layout=True, cmap='plasma')
hs.plot.plot_spectra(component_list,legend=legend_list)
diff_components = component_list[1].isig[E_range[0]:E_range[1]]/component_list[1].isig[E_range[0]].data[0]-component_list[0].isig[
diff_components.metadata.General.title = 'Difference components 1 minus 0 -'
diff_components.plot()

E_vector = np.linspace(E_range[0],E_range[1],1+int((E_range[1]-E_range[0])/hl.axes_manager['Energy loss'].scale))

df.plot()
spat_scale = df.axes_manager['x'].scale
m0 = hs.plot.markers.rectangle(x1=(roi_list[0][2]-0.5)*spat_scale, x2=(roi_list[0][3]+0.5)*spat_scale, y1=(roi_list[0][0]-0.5)*spa
m1 = hs.plot.markers.rectangle(x1=(roi_list[1][2]-0.5)*spat_scale, x2=(roi_list[1][3]+0.5)*spat_scale, y1=(roi_list[1][0]-0.5)*spa
df.add_marker(m0)
df.add_marker(m1)
```

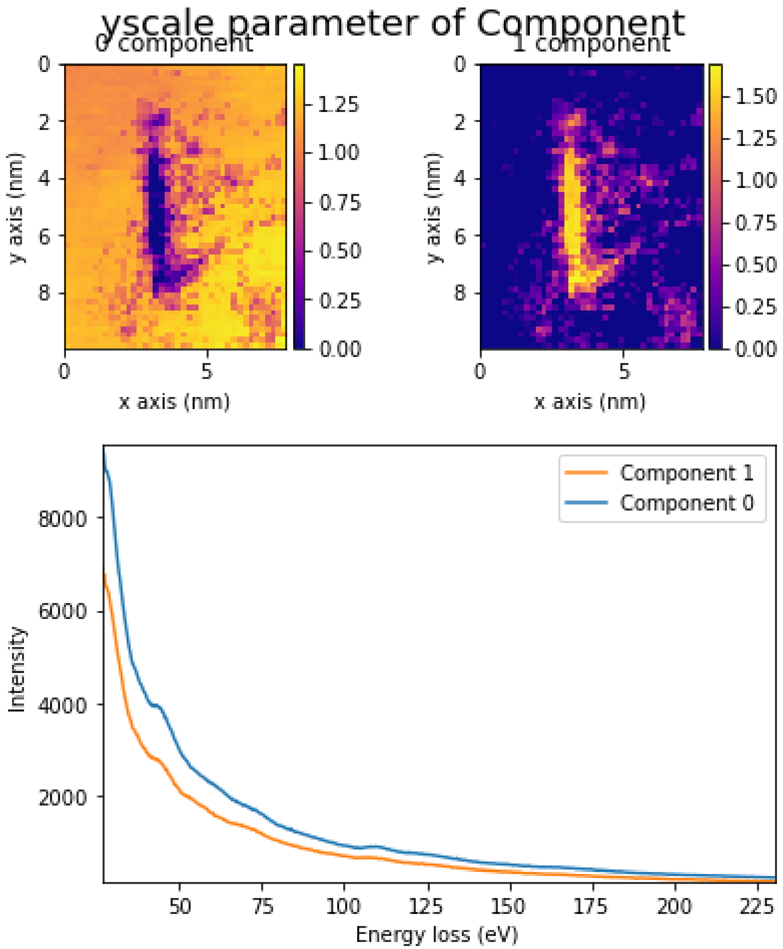
yscale parameter of Component
0 component
1 component
y axis (nm)
x axis (nm)
0
2
4
6
8
5
1.50
1.25
1.00
0.75
0.50
0.25
0.00
Component 1
Component 0
Intensity
8000
6000
4000
2000
50
75
100
125
150
175
200
225
Energy loss (eV)

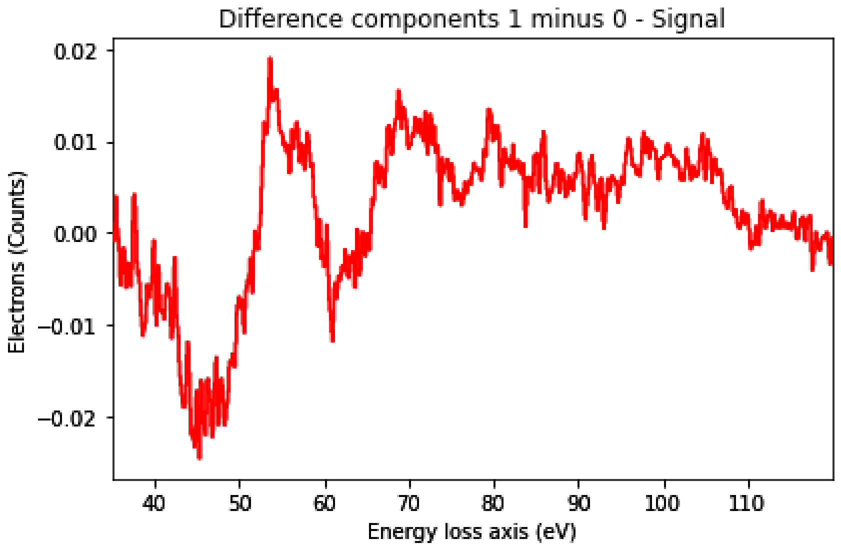
Difference components 1 minus 0 - Signal
0.02
0.01
0.00
−0.01
−0.02
Electrons (Counts)
40
50
60
70
80
90
100
110
Energy loss axis (eV)

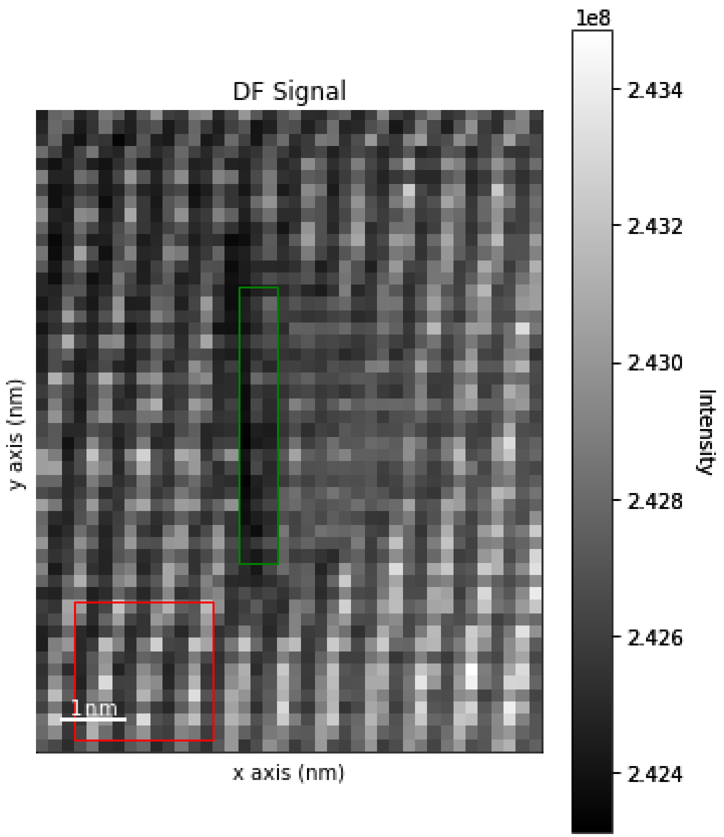
DF Signal
y axis (nm)
1nm
x axis (nm)
1e8
2.434
2.432
2.430
2.428
2.426
2.424
Intensity

Create the finialized figure with the subfigures collected. Here the data is also rotated 90 deg to match the displayed pyramids in the rest of the images.

a) The overview image with rectangle of the location of the spectrum image is displyed. Scalebar size is calculated and displayed

b) The image (same as above) is shown with the ROIs marked in correct colors. Also here a scalebar is shown.

c) The two fitted maps are shown as rgb-images (their intensity times the "color_components")

d) Finally the components shown above are plotted normalized to the max-value of each of them in the range (for comparison). The max-value is the first in each of them. Below this plot the difference is displayed with zero marked with a red line.

(Total figure is then saved for inclusion in the manuscript)

In [8]:

```
#Define colors to use for the components
color_components = [(0, 0.8, 0.8),(0.8, 0.8, 0)]

#Define size of figure
fig = plt.figure(frameon=False, figsize=(86*2/25.4, 70/25.4))
outer = gridspec.GridSpec(1, 3, wspace=0.2, width_ratios=[4,3,3])

#subfigure a
inner = gridspec.GridSpecFromSubplotSpec(1, 1, subplot_spec=outer[0], wspace=0, hspace=0)
ax = plt.Subplot(fig, inner[0])
ax.imshow(ndimage.rotate(survey, -90), cmap='gray')
scalebarsize = 5 #in nm
corrected_size = scalebarsize/survey.axes_manager['x'].scale
color_scalebar = 'black'
fontprops = fm.FontProperties(size=10,weight='bold')
scalebar = AnchoredSizeBar(transform=ax.transData, size=corrected_size, label=str(scalebarsize) + ' nm', loc=3, frameon=True, colo
rect = patches.Rectangle((240, 350), 490, 370, linewidth=2, edgecolor='r', facecolor='none')
ax.add_artist(scalebar)
ax.add_patch(rect)
ax.set_axis_off()
fig.add_subplot(ax)

#subfigure b
inner = gridspec.GridSpecFromSubplotSpec(2, 1, subplot_spec=outer[1], wspace=0, hspace=0, height_ratios=[2,1])
ax = plt.Subplot(fig, inner[0])
ax.imshow(ndimage.rotate(df, -90), cmap='gray')
scalebarsize = 2 #in nm
```

```python
corrected_size = scalebarsize/df.axes_manager['x'].scale
color_scalebar = 'black' #change if image to bright
fontprops = fm.FontProperties(size=10,weight='bold')
scalebar = AnchoredSizeBar(transform=ax.transData, size=corrected_size, label=str(scalebarsize) + ' nm', loc=3, frameon=True, colo
#show the locations of the components. Adjustments done to match rectangle after rotation
rect1 = patches.Rectangle((df.data.shape[0]-roi_list[0][1]-0.5, roi_list[0][2]-0.5), roi_list[0][1]-roi_list[0][0], roi_list[0][3]
rect2 = patches.Rectangle((df.data.shape[0]-roi_list[1][1]-0.5, roi_list[1][2]-0.5), roi_list[1][1]-roi_list[1][0], roi_list[1][3]
ax.add_artist(scalebar)
ax.add_patch(rect1)
ax.add_patch(rect2)
ax.set_axis_off()
fig.add_subplot(ax)

#subfigure c
inner2 = gridspec.GridSpecFromSubplotSpec(1, 2, subplot_spec=inner[1], wspace=0, hspace=0)
ax = plt.Subplot(fig, inner2[0])
image_list[0] = image_list[0]/image_list[0].max().data[0]
rgb_version1 = np.dstack((image_list[0],image_list[0],image_list[0]))
rgb_version1[:,:,0] = rgb_version1[:,:,0]*color_components[0][0]
rgb_version1[:,:,1] = rgb_version1[:,:,1]*color_components[0][1]
rgb_version1[:,:,2] = rgb_version1[:,:,2]*color_components[0][2]
rgb_version1 = (rgb_version1 * 255).astype(np.uint8)
ax.imshow(ndimage.rotate(rgb_version1, -90))
ax.set_axis_off()
fig.add_subplot(ax)
ax = plt.Subplot(fig, inner2[1])
image_list[1] = image_list[1]/image_list[1].max().data[0]
rgb_version2 = np.dstack((image_list[1],image_list[1],image_list[1]))
rgb_version2[:,:,0] = rgb_version2[:,:,0]*color_components[1][0]
rgb_version2[:,:,1] = rgb_version2[:,:,1]*color_components[1][1]
rgb_version2[:,:,2] = rgb_version2[:,:,2]*color_components[1][2]
rgb_version2 = (rgb_version2 * 255).astype(np.uint8)
ax.imshow(ndimage.rotate(rgb_version2, -90))
ax.set_axis_off()
fig.add_subplot(ax)

#subfigure d
inner = gridspec.GridSpecFromSubplotSpec(2, 1, subplot_spec=outer[2], wspace=0, hspace=0)
ax = plt.Subplot(fig, inner[0])
ax.plot(E_vector,component_list[0].isig[E_range[0]:E_range[1]].data/component_list[0].isig[E_range[0]].data[0],label='Mat (M)',col
ax.plot(E_vector,component_list[1].isig[E_range[0]:E_range[1]].data/component_list[1].isig[E_range[0]].data[0],label='Int (I)',col
ax.legend(frameon=False)
ax.axes.xaxis.set_ticklabels([])
ax.set_yticklabels(ax.get_yticks(), rotation=90)
```

```
fig.add_subplot(ax)
ax = plt.Subplot(fig, inner[1])
ax.axhline(y=0, color='r', linestyle='-')
ax.plot(E_vector,component_list[1].isig[E_range[0]:E_range[1]].data/component_list[1].isig[E_range[0]].data[0]-component_list[0].i
ax.legend(frameon=False)

ax.set_yticklabels(ax.get_yticks().round(decimals=2), rotation=90)
fig.add_subplot(ax)

#Change the tickmarks to include 'eV' as the unit
ax.set_xticklabels(['','50','100 eV',''])


#subfigure labels
fig.text(0.1, 0.77, 'a)', fontsize=10,fontweight='bold')
fig.text(0.42, 0.77, 'b)', fontsize=10,fontweight='bold')
fig.text(0.42, 0.29, 'c)', fontsize=10,fontweight='bold')
fig.text(0.7, 0.51, 'd)', fontsize=10,fontweight='bold')

#show and save figure as png
fig.show()
dpi_val = 300
fig.savefig('MLLS_EELS.png', dpi=dpi_val, pad_inches=0, transparent=True, bbox_inches='tight')
```

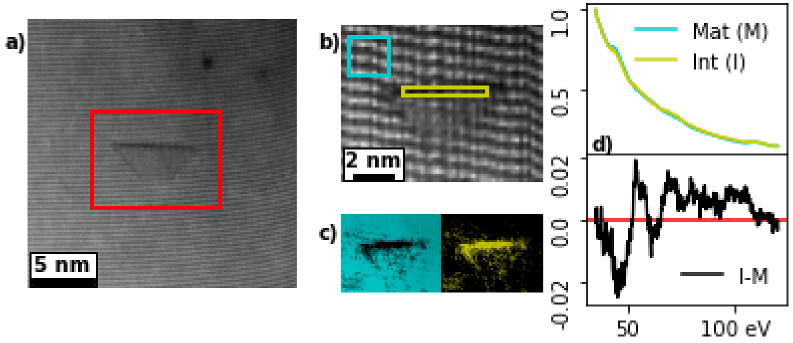

In [ ]:
